# A comparative study of the anthropometric status of adults and children in urban and rural communities of the North West Region of Cameroon

**DOI:** 10.1186/s40795-023-00734-9

**Published:** 2023-07-07

**Authors:** Faith Asangha Akob, Kirthee Pillay, Nicola Wiles, Muthulisi Siwela

**Affiliations:** 1 Dietetics and Human Nutrition, School of Agricultural, Earth and Environmental Sciences, University of KwaZulu-Natal, Private Bag X01, Scottsville 3209, Pietermaritzburg, 3201 South Africa; 2 School of Health and Medical Sciences, Catholic University of Cameroon, (CATUC), P.O Box 782, Mankon Bamenda, Cameroon

**Keywords:** Malnutrition, Obesity, Anthropometric status, Urban, Rural, Cameroon

## Abstract

**Background:**

Cameroon, like many other developing countries, is experiencing a double burden of malnutrition. With increasing urbanization, communities are exposed to high calorie diets and sedentary lifestyles, which contribute to overnutrition. However, the nutritional status of the communities may vary with geographic location. The aim of the current study was to investigate the prevalence of underweight, overweight and abdominal obesity among adults as well as overweight, underweight, stunting and wasting among children in selected urban and rural communities of the North West Region (NWR) of Cameroon. The study also compared these parameters between selected urban and rural areas.

**Methods:**

Cross-sectional study design was used to investigate the anthropometric status of adults (18–65 years) and children (1–5 years) from two rural (Mankon and Mendakwe) and two urban (Mankon and Nkwen) communities in the NWR of Cameroon. The study included 156 adults and 156 children per study site from different households. A multistage sampling technique was used to select the participants and study sites Anthropometric measurements were taken using standardised methods for selected indices: weight, height, waist circumference and mid-upper arm circumference (MUAC). Data were analyzed using Statistical Package for the Social Sciences (SPSS) version 25 and a *p*-value of < 0.05 was considered statistically significant.

**Results:**

Adults from Nkwen (urban) were either overweight (*n* = 74; 47.4%) or obese (*n* = 44; 28.2%) with 43.6% (*n* = 68) from urban Mankon obese, whilst adults from rural Mankon were normal weight (49.4%; *n* = 77), 2.6% (*n* = 4) from Mendakwe (rural) were underweight and 64.1% (*n* = 100) were normal weight. Children in the rural areas were severely underweight (*n* = 45; 14.4%), while children in the urban areas were either normal (*n* = 158; 50.6) or overweight (*n* = 43; 13.8%). More females in the urban sites (*n* = 39; 53.4% in Nkwen and *n* = 43; 69.4% in urban Mankon) had a large waist circumference (WC) compared to those in the rural sites (*n* = 17; 22.1% in Mendakwe and *n* = 24; 38.1% in rural Mankon). Males in the urban areas had large WC compared to those in the rural sites (*n* = 19; 24.4% in Nkwen; *n* = 23; 24.7% in urban Mankon; *n* = 15; 16.1% in rural Mankon and *n* = 2; 2.6% in Mendakwe). Mid-upper arm circumference (MUAC) values indicated that most children in both urban (*n* = 147; 94.2% in Nkwen; *n* = 152; 97.4% in urban Mankon) and rural areas (*n* = 142; 91.0% in rural Mankon; *n* = 154; 98.7% in Mendakwe) were not acutely malnourished.

**Conclusions:**

This study found a higher prevalence of overweight and obesity among adults and children in the urban areas of Nkwen and Mankon, compared to rural Mankon and Mendakwe. Thus, there is a need to investigate and address the causes of the high prevalence of overweight and obesity in these urban areas.

**Supplementary Information:**

The online version contains supplementary material available at 10.1186/s40795-023-00734-9.

## Background

Malnutrition, including both under and overnutrition are serious health concerns in developing countries like Cameroon. In Cameroon, the double burden of malnutrition mainly presents as undernutrition, in the form of underweight, protein energy malnutrition (PEM) and micronutrient deficiencies, and overnutrition in the form of overweight, obesity and obesity-related diseases [1]. A proper diet, in terms of quality and quantity, has a significant impact on nutritional status and plays an important role in the health and development of an individual [2]. However, consuming inadequate quantities of food with poor nutrient quality results in malnutrition [2]. Anthropometry has long been used to assess nutritional status as this is an inexpensive non-invasive method that provides detailed information on different components of body structure [3]. The core elements of anthropometry are weight, height, body mass index (BMI) and body circumference measurements [4]. These measurements are important because they represent diagnostic criteria for obesity, which significantly increases the risk for non-communicable diseases (NCDs) like type 2 diabetes, hypertension, cardiovascular disease and some cancers [4].

In Cameroon, the focus of the government and non-governmental organizations (NGOs) has always been on undernutrition rather than overnutrition [5, 6], especially in some of the French regions of the country, for example the Far North, Adamawa and East. Generally, communities in these regions cannot afford a nutritious diet due to the ongoing Boko Haram war, which has contributed to poverty [6]. On the other hand, increased urbanization in Cameroon has caused a nutrition transition characterized by high calorie diets and sedentary lifestyles [7], leading to an increase in NCDs [3]. According to the 2018 Global Nutrition Report, the prevalence of overweight (BMI ≥ 25 kg/m^2^) and obesity (BMI ≥ 30 kg/m^2^) among Cameroonian women 18 years and older in 2016 was 41.7% and 16.4%, respectively, which is an increase from 2014 of 40.2% and 15.4%, respectively [8]. Among adult men, 25.5% were overweight and 6.1% were obese in 2016, which is an increase from 2014 of 24% and 5.5%, respectively [8]. In addition, 6.5% of adult men and 6.9% of women of reproductive age were diagnosed as diabetic in 2014, with a projected increase to 7.7% and 8.3%, respectively in 2019 [8]. Hypertension (blood pressure above 140/90 mm/Hg) was more prevalent in women 16 years and above (24.9%), compared to men (24.6%). In addition, high low-density lipoprotein (LDL) levels (higher than 100 mg/dl) was observed in 20% and 24% of adult men and women, respectively [9, 10]. The prevalence of stunting in Cameroonian children under the age of five years in 2014 was 31.7%, which decreased to 29% in 2018. In 2014, 5.2% of Cameroonian children under the age of five years were wasted, which decreased to 4.3% in 2018 [9, 10]. However, in 2014, 6.7% were overweight and this increased to 11.0% in 2018 [9, 10].

It is evident that both under and overnutrition exist in Cameroon. However, nutrition data from specific regions and target groups within the regions are required to implement targeted nutritional interventions. The available literature indicates that no detailed studies on the anthropometric status of specific population groups from the urban and rural areas of the North West Region (NWR) of Cameroon have been conducted. To address the lack of data, the current study aimed to investigate the prevalence of underweight, overweight and abdominal obesity among adults as well as overweight, underweight, stunting and wasting among children in selected urban and rural communities of the NWR of Cameroon. The study also compared these parameters between selected urban and rural areas.

## Methods

a Study design

Cross-sectional study design was used to investigate the anthropometric status of adults and children of selected rural and urban communities in the NWR of Cameroon.

b Sample size and sample selection

A research statistician used an online sample size calculator to calculate the sample size [11]. To get the results that reflected the target population in terms of gender and age as precisely as needed, a 95% confidence interval (1.96 confidence interval of a population of 2,180,309) in the NWR was assumed and a 5% margin error [12]. In addition, a power analysis for a chi-square test indicated that the minimum sample size needed to yield a statistical power of at least 0.8 with an alpha of 0.5 and medium effect size of 0.5 was 601, based on the population size [11]. The study sample was 1248 adults and children, which was above the minimum 601; 624 children and 624 adults per the two rural and two urban areas. Therefore, 156 children and 156 adults (total = 312) per study area were required. These study areas were chosen because no similar nutrition studies have been carried out in these areas and it has been suggested that more nutrition research should be carried out in Cameroon [13]. The study areas were randomly selected using the simple random sampling procedure. With this procedure, random numbers between 1 and 10 were generated and allocated to all seven council areas within Mezam division. Small papers with the numbers and council areas written on them were put in a box and four papers were randomly picked to select the four study areas. Stratified random sampling was used to recruit study participants. Households that had adults (18–65 years) and children (1–5 years) were identified and listed for each study area. Adolescents of 18–19 years were merged with the adult population because most studies carried out in Cameroon consider persons 18 years and above to be adults. This categorisation of adults and children, respectively, is also found in the Global Nutrition Report 2021 [8].

Every odd numbered household that had adults and children who met the inclusion criteria were allowed to participate independently of the other members of the population. Children (1–5 years) were only included in the study if their caregiver gave consent. Adults from the target age group of 18–65 years old were chosen because they are at increased risk for developing NCDs [9]. Children in the age group of 1–5 years were chosen because at this stage they grow and develop fast, their eating behavior evolves and they are vulnerable to malnutrition [14]. Adults and children who did not fall within the target group at the time of data collection were excluded. Pregnant women were excluded because it is well accepted that BMI is not used during pregnancy [15]. All individuals who were invited to participate in the study, agreed to participate.

c Pilot study

A pilot study was conducted on 10% of the sample population [16] (10% of 2496 = 250 participants), which equated to 125 adults and 125 children. The study was conducted in Mbengwi, an area out of the municipality that was not to be used in the main study. The purpose of the pilot study was to investigate whether the data collection sheet was appropriate for the target group, to investigate the time required to take anthropometric measurements and establish whether or not field workers were using the correct procedures to take the anthropometric measurements. The pilot study found that some field workers were not allowing the scale to read zero before taking each weight measurement. This was corrected before the main data collection. The pilot study participants were excluded from the main study.

d Anthropometric data collection

Anthropometric data was recorded by the fieldworkers using a Questionnaire/Data collection sheet (Supplementary file 1). The field workers, who were recruited from National Polytechnic University Institute (NPUI) Bamenda, were trained by the researcher to take and record the anthropometric measurements. The data collection sheet was developed in English, as it is the language spoken in the chosen study areas. Cameroon is a bilingual country with two official languages, English and French, and more than 1 000 tribal languages. Anglophone (English speaking) study areas were used, where English is spoken and understood by all [5]. Therefore, the field workers and study participants could all understand English.

The WHO Steps Surveillance and Centers for Disease Control (CDC) procedures on how to take the different anthropometric measurements was used [17, 18]. Weight, height and WC measurements were taken from adults (18–65 years old), while weight, height, and MUAC measurements were taken from children (1–5 years old). The EatSmart Precision Plus Digital bathroom weight scale (EatSmart Chicago, USA) was used to take weight measurements in kilograms (kg), while an HM200P PortStad portable stadiometer (Charder, USA) was used to take height measurements in centimetres (cm) and converted to meters (m) for adults and children above two years of age. A Seca 210 baby length measuring mat was used to take height measurements in children below two years old (Seca Quick Medicals, USA). A non-stretch fibre glass measuring tape (S0145620, UNICEF, USA) was used to take MUAC measurements in children 1–5 years in cm, while a circumference diameter tape (Wintape Measuring Tape Co., Ltd Guanghou, China) was used to measure WC in adults (18–65 years). All anthropometric measurements were taken three times and read to the nearest one decimal place and the mean values of three readings were calculated and recorded.

### Adults

#### Weight

The scale was calibrated at the start of the day before any measurements were taken and also at the end of the day when all measurements had been taken. The scale was zeroed before and after each participant was weighed. Participants removed their shoes before being weighed and stood in the middle of the scale with their feet together and as still as possible. Participants were weighed with minimal clothing and with no heavy items in their pockets.

#### Height

Participants removed their shoes, hair wigs and hair accessories before height measurements were taken. They stood with their backs to the wall where the equipment was placed and the head was positioned in the Frankfort plane [18]. They were positioned so that their feet, calves, bottom, upper back and the back of their head were in contact with the back of the stadiometer, directly underneath the head piece measuring device. The field worker lowered the measuring device until it rested on top of the participant’s head and the measurements were recorded to the nearest 0.1 cm.

#### Body mass index (BMI)

Mean weight and height values were used to calculate the BMI for each adult participant using the following equation [10]:

$$Body\;mass\;index=\frac{Weight(kg)}{Height{(m)}^2}$$

Body mass index was used to classify the weight status of adults, using the international classification of adult underweight, overweight and obesity [10].

#### Waist circumference (WC)

Participants were asked to remove all heavy clothing before WC measurements were taken. Field workers took WC measurements at the umbilicus (at the navel) for all adult participants. The measuring tape was placed in a horizontal plane around the abdomen at the level of the iliac crest. Before reading the tape measure, the field workers ensured that the tape was snug and did not compress the skin. A WC above 80 cm and 94 cm for females and males, respectively indicates risk for comorbidities, including obesity, while a WC above 88 cm and 102 cm for females and males, respectively indicates increased risk for comorbidities, including obesity [19]. This classification was used to investigate the risk for comorbidities in this study.

### Children

#### Weight

The scale was calibrated at the start of the day before any measurements were taken and at the end of the day when all measurements had been taken. The scale was zeroed before and after each child was weighed. The children were weighed with clean, dry diapers and minimal clothing. The diaper or napkin worn by children from rural areas was felt from the outside to check if it was wet and heavy. If it was wet and heavy, the caregiver was asked to change the diaper or napkin before the weight measurements were taken. The WHO cut-offs (WHO Child Growth Standards 2012) were used to classify weight-for-age and weight-for-height in this study [14].

#### Height/Length

The height of children below two years old was measured using a Seca 210 baby length measuring mat. The mat was placed on a level floor before the measurements were taken. The caregiver removed the child’s shoes and any other heavy clothing before the child was placed on the mat. One fieldworker held the child’s head in a straight position against the board, while another fieldworker used one hand to hold the child’s legs down without bending the knees and the other hand to move the foot board towards the child’s heels. The procedure used for taking height measurements in adults was also used for children two years and older.

#### Mid-upper arm circumference (MUAC)

The MUAC measurement was taken halfway between the acromion process and the tip of the olecranon process of the left arm of all child participants. Fieldworkers made sure that the arm was relaxed and the tape was not too tight or loose when the measurements were taken. Measurements were recorded to the nearest 0.1 cm. Measurements were taken and a mean was calculated and classified using the WHO guidelines [20].

e Data analysis

Data were entered and captured onto Microsoft (MS) Excel spreadsheets by the researcher and then transferred to Statistical Package for the Social Sciences (SPSS) version 25 (SPSS Inc., Chicago, IL, USA) for statistical analysis by a statistician. Data were analyzed using descriptive statistics including means, standard deviations and frequencies. A chi-square test was used to investigate relationships among categorical variable responses selected (anthropometric data and study areas). An analysis of variance (ANOVA) test was used to compare two or more groups of cases in one variable (WC for males and females from different study areas). The Fisher’s Exact and Welch tests were used when statistical conditions were not met. A *p*-value of < 0.05 was taken as statistically significant.

f Ethics approval and consent to participate

All methods in this study were carried out in accordance with the Declaration of Helsinki Ethical Principles for Medical Research Involving Human Subjects. Ethical approval was obtained from the Biomedical Research Ethics Committee of the University of KwaZulu-Natal, South Africa (Reference number: BE439/19) and the Regional Hospital Institutional Review Board Cameroon (Reference number: 072/APP/RDPH/RHB/IRB). Gate keeper’s permission was obtained from the Regional Delegation of Health North West Region Cameroon (Reference number: 52/ATT/NWR/RDPH). Informed and signed consent was obtained from all adult participants and the caregivers of child participants. Permission was obtained from the National Polytechnic University Institute to use some degree level nutrition students as field workers.

## Results

### Demographic characteristics

The demographic characteristics (age and gender) of the adult participants per study area are presented in Table 1.

**Table 1:** Age ranges and gender of adult participants (18–65 years old) per study area (*n* = 156)

Area	Age group (males)	n (%) ^a^	Age group (females)	n (%) ^a^
Nkwen (urban) (*n* = 156)	18 years	0 (0)	18 years	1 (0.6)
	19–30 years	14 (9.0)	19–30 years	40 (25.6)
	31–50 years	62 (39.7)	31–50 years	28 (17.9)
	51–65 years	2 (1.3)	51–65 years	4 (2.6)
Mankon (urban) (*n* = 156)	18 years	0 (0)	18 years	0 (0)
	19–30 years	37 (23.7)	19–30 years	38 (24.4)
	31–50 years	56 (35.9)	31–50 years	23 (14.7)
	51–65 years	0 (0)	51–65 years	1 (0.6)
Mankon (rural) (*n* = 156)	18 years	0 (0)	18 years	1 (0.6)
	19–30 years	21 (13.5)	19–30 years	18 (11.5)
	31–50 years	59 (37.8)	31–50 years	36 (23.1)
	51–65 years	13 (8.3)	51–65 years	19 (12.2)
Mendakwe (rural) (*n* = 156)	18 years	0 (0)	18 years	1 (0.6)
	19–30 years	23 (14.7)	19–30 years	19 (12.2)
	31–50 years	58 (37.2)	31–50 years	45 (28.8)
	51–65 years	3 (1.9)	51–65 years	12 (7.7)
TOTAL		348 (55.8)		276 (44.2)

^a^Percentage of total sample per area (*n* = 156)

More than half of the adult study participants were males (*n* = 348; 55.8%) between the ages of 31 and 50 years old in all study areas.

Table 2 shows that more than half of the child study participants were females (*n* = 366; 58.7%) between the ages of 1–3 years in all areas.

**Table 2:** Age ranges and gender of children (1–5 years old) per study area (*n* = 156)

Area	Age group (males)	n (%) ^a^	Age group (females)	n (%) ^a^
Nkwen (urban) (*n* = 156)	1–3 years 4–5 years	33 (21.2) 25 (16.0)	1–3 years 4–5 years	67 (42.9) 31 (19.9)
Mankon (urban) (*n* = 156)	1–3 years 4–5 years	48 (30.8) 5 (3.2)	1–3 years 4–5 years	92 (59.0) 11 (7.1)
Mankon (rural) (*n* = 156)	1–3 years 4–5 years	63 (40.4) 7 (4.5)	1–3 years 4–5 years	70 (44.9) 16 (10.3)
Mendakwe (rural) (*n* = 156)	1–3 years 4–5 years	38 (24.4) 39 (25.0)	1–3 years 4–5 years	44 (28.2) 35 (22.4)

^a^Percentage of total sample per area (*n* = 156)

## Anthropometry

### Adults

#### Body mass index (BMI)

Table 3 shows BMI classification by gender. A chi-square Fisher’s Exact test showed no significant relationship between BMI and gender.

**Table 3:** Body mass index classification for adults 18–65 years old by gender (*N* = 624)

BMI classification	Males n (%) ^a^	Females n (%) ^a^	Total n (%) ^a^	*P* value^#^
Underweight (< 18.5 kg/m^2^)	3 (0.5)	3 (0.5)	6 (1.0)	0.156
Normal (18.5–24.9 kg/m^2^)	142 (22.8)	113 (18.1)	255 (40.9)	
Overweight (25.0–29.9 kg/m^2^)	111 (17.8)	106 (17.0)	217 (34.8)	
Obese (≥ 30.0 kg/m^2^)	92 (14.7)	54 (8.7)	146 (23.4)	

^#^Chi-square test, *p* values in bold are statistically significant

^a^Percentage of total sample (*N* = 624)

Table 4 shows BMI classification for adults by study area.

**Table 4:** Body mass index classification for adults 18–65 years by area (*N* = 624)

BMI classification					
**Area**	**Underweight (< 18.5 kg/m**^**2**^**) n (%) **^**a**^	**Normal (18.5–24.9 kg/m**^**2**^**) n (%) **^**a**^	**Overweight (25.0–29.9 kg/m**^**2**^**) n (%) **^**a**^	**Obese (≥ 30.0 kg/m**^**2**^**) n (%) **^**a**^	***P* value**^**#**^
Nkwen (urban) (*n* = 156)	1 (0.6)	37 (23.7)	74 (47.4)	44 (28.2)	** < 0.001**
Mankon (urban) (*n* = 156)	0 (0)	41 (26.3)	47 (30.1)	68 (43.6)	
Mankon (rural) (*n* = 156)	1 (0.6)	77 (49.4)	55 (35.3)	23 (14.7)	
Mendakwe (rural) (*n* = 156)	4 (2.6)	100 (64.1)	41 (26.3)	11 (7.1)	

*BMI* Body mass index

^#^Chi-square test, *p* values in bold are statistically significant

^a^Percentage of total sample per area (*n* = 156)

A chi-square test of independence showed that the urban areas had more overweight and obese participants than the rural areas. The results show that a significant number of adults from Nkwen (urban) were either overweight (*n* = 74; 47.4%) or obese (*n* = 44; 28.2%) with 43.6% (*n* = 68) from urban Mankon obese. A significant number of adults from rural Mankon were normal weight (49.4%; *n* = 77), while 2.6% (*n* = 4) from Mendakwe (rural) were underweight and 64.1% (*n* = 100) were normal weight (Table 4).

#### Waist circumference (WC)

Table 5 shows the classification of WC by area for adult males and females.

**Table 5:** Waist circumference classification for adults (18–65 years) across all four study areas (*N* = 617)

**WC classification**									
**Females (*n* = 275)**^d^					**Males (*n* = 342)**^d^				
**Area**	**Normal (< 80 cm) n (%)**^c^	**At risk**^a^**(80–88 cm) n (%)**^c^	**Increased risk**^b^**(> 88 cm) n (%)**^c^	***P* value**^**#**^	**Area**	**Normal (< 94 cm) n (%)**^c^	**At risk**^a^**(94–102 cm) n (%)**^c^	**Increased risk**^b^** (> 102 cm) n (%)**^c^	***P* value**^**#**^
Nkwen (urban) (*n* = 73)	13 (17.8)	21 (28.8)	39 (53.4)	** < 0.0005**	Nkwen (urban) (*n* = 78)	53 (67.9)	19 (24.4)	6 (7.7)	** < 0.0005**
Mankon (urban) (*n* = 62)	10 (16.1)	9 (14.5)	43 (69.4)		Mankon (urban) (*n* = 93)	57 (61.3)	23 (24.7)	13 (14.0)	
Mankon (rural) (*n* = 63)	18 (28.6)	21 (33.3)	24 (38.1)		Mankon (rural) (*n* = 93)	68 (73.1)	15 (16.1)	10 (10.8)	
Mendakwe (rural) (*n* = 77)	38 (49.4)	22 (28.6)	17 (22.1)		Mendakwe (*n* = 78)	76 (97.4)	2 (2.6)	0 (0)	

*WC* Waist circumference

^#^Welch test, *p* values in bold are statistically significant

^a^Increased risk for obesity and related diseases

^b^Substantially increased risk for obesity and related diseases

^c^Percentage of total male/female per urban/rural area

^d^n does not equal to 276 for females and 348 for males due to missing data

The Welch Robust test of equality of means indicated that there were significant relationships between WC and area (*p* < 0.0005). Generally, in all areas, females had a larger WC than males. A significant number of adult females in the urban areas of Nkwen (urban) (*n* = 39; 53.4%) and urban Mankon (*n* = 43; 69.4%) had larger WC (greater than 88 cm) than males in Nkwen (urban) (*n* = 6; 7.7%) and urban Mankon (*n* = 13; 14.0%), putting them at substantially increased risk for obesity and related diseases. In addition, a significant number of adult females and males in the rural areas of Mendakwe and rural Mankon had normal WC.

### Children

#### Weight-for-height (WFH), weight-for-age (WFA) and height-for-age (HFA) classification

Table 6 shows the WFH, WFA and HFA classification for children 1–5 years in the urban and rural areas.

**Table 6:** Weight-for-height, weight-for-age and height-for-age classification for children in the urban and rural areas (*N* = 624)

Weight-for-height			Weight-for-age			Height-for-age		
**Classification**	**n (%) **^**a**^	***P* value**^**#**^	**Classification**	**n (%) **^**a**^	***P* value**^**#**^	**Classification**	**n (%) **^**a**^	***P* value**^**#**^
**Urban areas (*n* = 312)**								
Severely wasted	3 (1.0)	0.165	Severely underweight	26 (8.3)	** < 0.001**	Severely stunted	151 (48.4)	** < 0.001**
Moderately wasted	9 (2.9)		Moderately underweight	85 (27.2)		Moderately stunted	42 (13.5)	
Normal	139 (44.6)		Normal	158 (50.6)		Normal	56 (17.9)	
Overweight	161 (51.6)		Overweight	43 (13.8)		Tall	63 (20.2)	
**Rural areas (*n* = 312)**								
Severely wasted	6 (1.9)	0.165	Severely underweight	45 (14.4)	** < 0.001**	Severely stunted	135 (43.3)	** < 0.001**
Moderately wasted	6 (1.9)		Moderately underweight	115 (36.9)		Moderately stunted	53 (17.0)	
Normal	172 (55.1)		Normal	124 (39.7)		Normal	97 (31.1)	
Overweight	128 (41.0)		Overweight	28 (9.0)		Tall	27 (8.7)	

^#^Chi-square test, *p* values in bold are statistically significant

^a^Percentage of total sample per urban/rural area (*n* = 312)

Many children in the rural areas were severely underweight (*n* = 45; 14.4%) while most children in the urban areas were either normal (*n* = 158; 50.6%) or overweight (*n* = 43; 13.8%). In addition, most of children in the rural areas had normal height (*n* = 97; 31.1%) while a good number in the urban areas were tall (*n* = 63; 20.2%).

#### Mid-upper arm circumference classification

Table 7 shows the MUAC classification for children 1–5 years old by area.

**Table 7:** MUAC classification for children 1–5 years old by area (*n* = 156)

MUAC classification				
**Area**	**SAM (< 11.0 cm) n (%) **^**a**^	**MAM (11.5 cm-12.5 cm) n (%) **^**a**^	**NAM (> 12.5 cm) n (%) **^**a**^	***P* value**^**#**^
Nkwen (urban) (*n* = 156)	3 (1.9)	6 (3.8)	147 (94.2)	**0.014**
Mankon (urban) (*n* = 156)	0 (0)	4 (2.6)	152 (97.4)	
Mankon (rural) (*n* = 156)	2 (1.3)	12 (7.7)	142 (91.0)	
Mendakwe (rural) (*n* = 156)	0 (0)	2 (1.3)	154 (98.7)	

*MUAC* Mid-upper arm circumference, *SAM* Severe acute malnutrition, *MAM* Moderate acute malnutrition, *NAM* Not acutely malnourished

^#^Chi-square test, *p* values in bold are statistically significant

^a^Percentage of total sample per urban/rural area (*n* = 156)

Most of the children had normal MUAC in Nkwen (urban) (*n* = 147; 94%), urban Mankon (*n* = 152; 97%), rural Mankon (*n* = 142; 91.0%) and rural Mendakwe (*n* = 154; 98.7%). According to MUAC classification, a significant number of children had SAM in Nkwen (urban) (*n* = 3; 1.9%) and MAM in rural Mankon (*n* = 12; 7.7%) (Table 7).

## Discussion

The results of this study indicated that over and undernutrition are prevalent in the NWR of Cameroon. This is in keeping with results from the World Food Program (WFP), which also mentioned that over and undernutrition was prevalent in Cameroon [1]. The high prevalence of overweight and obesity among adults in urban Nkwen and urban Mankon in this study, is in line with a study on the assessment of nutritional status and food consumption conducted in Makepe-Missoke Douala, Cameroon, which indicated that overweight and obesity were prevalent in the urban adult female population [21]. In Africa, the high prevalence of overweight and obesity has been attributed to the nutritional transition that has resulted from urbanization and westernization of lifestyle behavior, which include poor dietary habits and sedentary lifestyles [22]. As mentioned earlier, the World Bank, stated that increased urbanization in Cameroon has caused a nutrition transition characterized by high calorie diets and lack of physical activity, which are possible reasons for the high prevalence of obesity among adults in urban Cameroon [7]. In addition, the results of this study showed that females generally had larger WC than men, which is in accordance with results from other studies [19, 23, 24]. Although there was an unequal gender distribution in the current study, which may have led to unintentional bias, female participants still had a larger WC than males. This difference in prevalence of overweight and obesity and larger WC between women and men have a biological basis [25]. Women have a higher percentage of body fat than men and their resting fat metabolism is lower than in men, leading to an increased risk for obesity [25]. The direct consequences of being overweight or obese and having a large WC are diabetes mellitus, cardiovascular disease, high blood pressure, osteoarthritis, dyslipidemia and cancer [24].

A study conducted in Cameroon, indicated low rates of overweight and obesity in rural areas, similar to the current study as most adults from rural Mankon and Mendakwe (rural) had a normal weight [26]. Another study conducted in Cameroon on physical activity and its relationship with obesity, high blood pressure and diabetes in urban and rural Cameroon, showed low physical activity practices in the urban areas, whereas in the rural areas, physical activity was practiced as most of the rural population worked on their farms [27]. Therefore, the possible reasons for the high prevalence of overweight and obesity in the urban areas as mentioned earlier, include poor dietary practices and a lack of physical activity. Given the consequences of overweight and obesity, it is time to address the factors causing the high prevalence of overweight and obesity among urban adults in the NWR of Cameroon.

Similar to the adults in the current study, undernutrition and overnutrition were also prevalent in children. In the last decade, undernutrition in children has received political and financial attention globally, including in Cameroon, while overnutrition in children is often overlooked and regarded as “healthy feeding” in most African countries [28]. Rural–urban differences in anthropometric status were observed among children under five years old in the current study. Nkwen and urban Mankon, which are urban, recorded more overweight children than underweight and normal weight children, compared to their rural counterparts. Meanwhile, Mendakwe (rural) and rural Mankon had more normal weight and undernourished children. This is in accordance with a study which found that in low- and middle-income countries, particularly the urban areas, the distribution of childhood nutritional diseases is shifting from undernutrition to overnutrition [28]. Another study on the challenges of underweight and overweight in South African children, showed that urban children are faced with a burden of overweight and obesity, while rural children are undernourished [29]. In addition, rural populations face a much higher burden of child undernutrition than urban populations in sub-Saharan Africa [30]. Most of the children in both the urban and rural study sites in the current study were not acutely malnourished with a mean MUAC > 12.5 cm, indicating a low prevalence of undernutrition. However, higher mean MUAC results were seen in children in the urban areas, compared to their rural counterparts. According to a study conducted similar to the current study, there was a direct relationship between child BMI, Z scores and MUAC, where the higher the BMI and Z scores, the higher the MUAC; most of the children in the urban areas were overweight [31]. Most of the children in urban areas have sedentary lifestyles, low physical activity levels and high consumption of high calorie foods and drinks [30, 32]. This could be a possible reason for the high prevalence of overweight and obesity among children in Nkwen and urban Mankon. Thus, there is a need to find the causes of the high prevalence of overweight and obesity among children as it increases their risk for developing NCDs later on in adulthood [8]. Children in rural areas are usually very active, either playing with friends, walking long distances to go to school or helping their parents on the farm, so they are at lower risk of being overweight [30]. In addition, the rural communities cannot afford to buy luxury food items such as sweets, biscuits and sweet fizzy drinks, which may explain why rural children consume less of these items compared to urban dwellers [30]. Most of the rural communities grow their own crops and rear animals and thus produce their own food [30]. This could be a possible reason for the low prevalence of overweight and higher prevalence of normal and underweight children in rural areas. Undernutrition has been linked to impaired cognitive development, reduced school attainment and slow economic growth [30]. Therefore, the causes of undernutrition in children in the rural areas need to be identified and addressed.

### Study limitations

The limitations of cross-sectional study design apply in the current study, including the fact that the possible changes in nutritional status with time were not investigated.

## Conclusion

The findings of the current study indicate that anthropometric status differed between urban and rural areas in Cameroon for both adults and children. Overweight and obesity were prevalent among adults (18–65 years) and children (1–5 years), particularly in the urban areas. The majority of adults and children in the rural areas were of normal weight, while only a few were underweight, stunted and wasted. Additionally, the urban population had more adults with a larger WC, especially females, as compared to their rural counterparts, placing them at increased risk for developing obesity-related diseases. However, it is important to note that there was an unequal gender distribution in this study, which may have led to the introduction of unintentional bias. The urban child population also had higher mean MUAC compared to the children in the rural areas, which when matched to anthropometric index classifications, puts the children at increased risk for developing obesity-related diseases later on in life. There is a need to investigate the causes of the high prevalence of overweight and obesity among adults and children in the urban areas of the NWR of Cameroon and to facilitate the formulation of intervention strategies to address the problem.

## Supplementary Information

**Additional file 1.**

## Data Availability

The data presented in this study are available on request from the first author (bfaithakob@yahoo.com).

## Abbreviations

NWR: North West Region
NGOs: Non-governmental Organizations
HDL: High-density Lipoprotein
LDL: Low-density Lipoprotein
WFP: World Food Program
WC: Waist Circumference
MUAC: Mid-Upper Arm Circumference
BMI: Body Mass Index
PEM: Protein-Energy Malnutrition
CDC: Centers for Disease Control and Prevention
USA: United States of America
