# Supplementary material for: A comparative study of the anthropometric status of adults and children in urban and rural communities of the North West Region of Cameroon
Source: BMC Nutr. 2023 Jul 7;9:81. doi: 10.1186/s40795-023-00734-9 (PMC10327329; doi:10.1186/s40795-023-00734-9)
Supplement: Supplementary file 1 — Additional file 1. [file 40795_2023_734_MOESM1_ESM.docx]

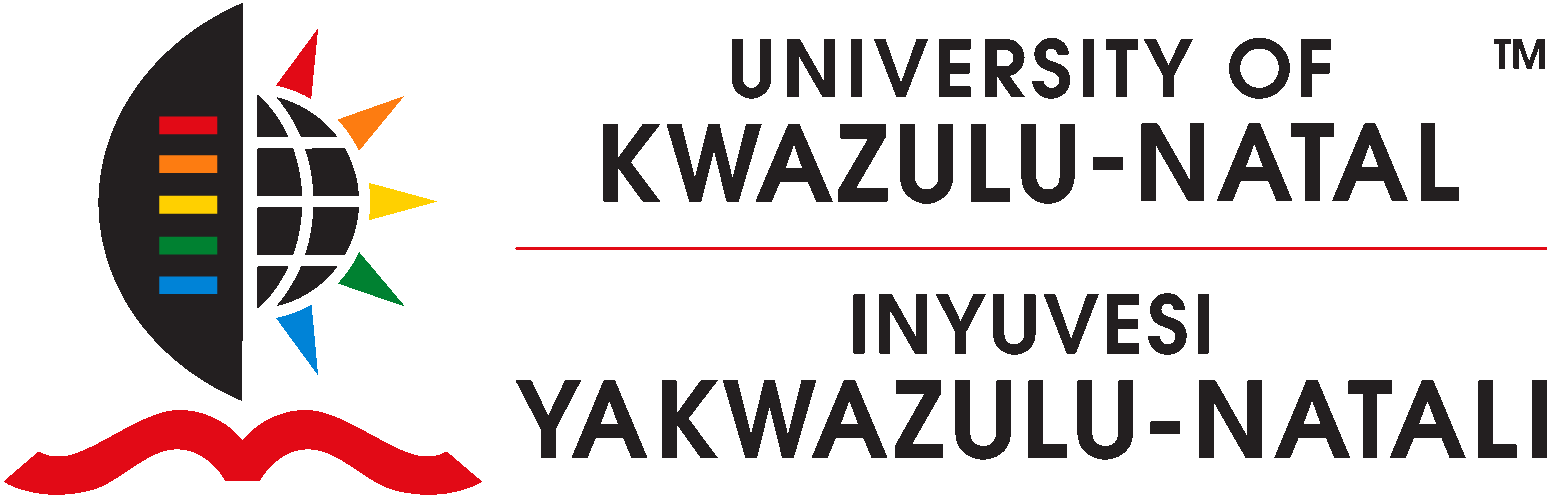
 **QUESTIONNAIRE / DATA COLLECTION SHEET FOR ADULTS**

**ANTHROPOMETRIC DATA (ADULTS)**

**Name of field worker:** __________________________________________

**Area/Area code:** _______________________________________________

**Urban/Rural:** ________________________________________

**Household Number/Participant number:** ___________________________

**Tick the correct response and give answers to the following questions**

1. How old are you? ____________years
2. Is there a history of diabetes, hypertension or obesity in your family?
3. YES, diabetes
4. YES, hypertension
5. YES, obesity
6. YES diabetes, hypertension and obesity
7. NO,
8. Have you been diagnosed with any chronic disease of lifestyle (example diabetes, high blood pressure or obesity?
9. YES which one/s? ______________________
10. No
11. For how long have you been diagnosed with a chronic disease of lifestyle (example diabetes, high blood pressure or obesity? ___________

Now we are going to measure your height, weight and waist to identify your body mass index and waist circumference. Do you want to continue with the assessments? (*Discontinue if participant refuses*)

**Weight and height should be measured three times and the scale must be calibrated before the measurements are taken.**

|  | **Age/**  **gender** | **Weight (Kilograms)** | | | | **Height (metres)** | | | | **Waist circumference (Centimetres)** | | | |
| --- | --- | --- | --- | --- | --- | --- | --- | --- | --- | --- | --- | --- | --- |
|  |  | 1st | 2nd | 3^rd^ | Mean | 1st | 2^nd^ | 3rd | Mean | 1st | 2nd | 3rd | Mean |
| Adult |  |  |  |  |  |  |  |  |  |  |  |  |  |

|  |  |  |  |  |  |  |  |  |  |  |  |  |  |  |  |  |  |
| --- | --- | --- | --- | --- | --- | --- | --- | --- | --- | --- | --- | --- | --- | --- | --- | --- | --- |
|  |  |  |  |  |  |  |  |  |  |  |  |  |  |  |  |  |  |

Interviewer’s signature: __________________ Date: _________________________


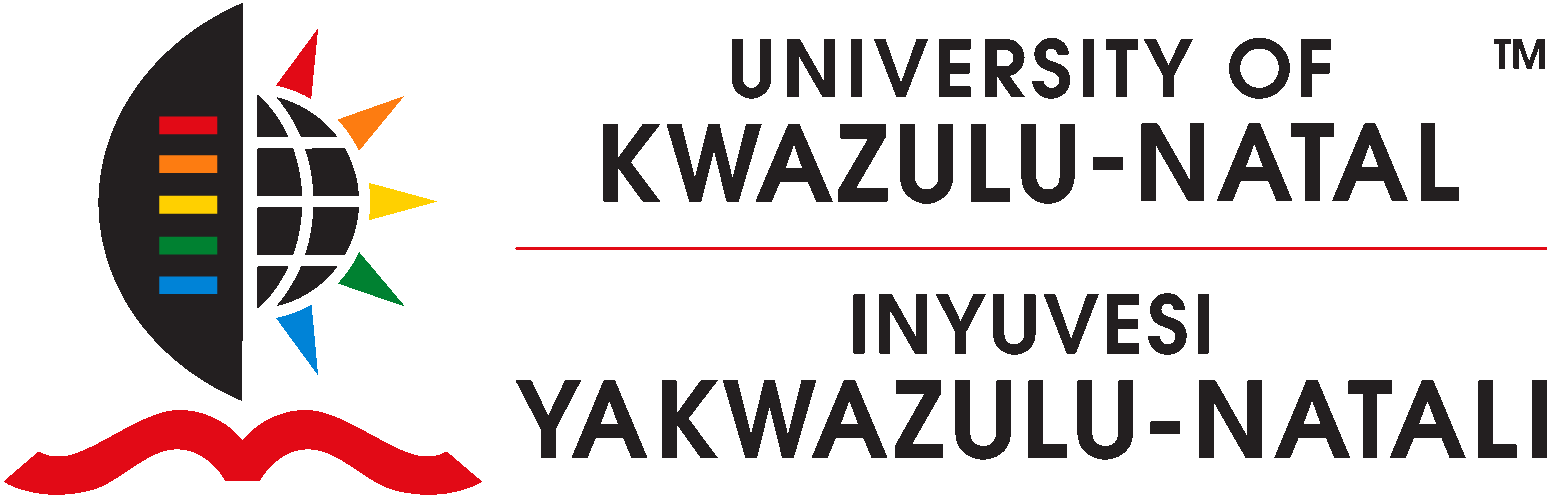
 **QUESTIONNAIRE / DATA COLLECTION SHEET FOR CHILDREN**

**ANTHROPOMETRIC DATA (CHILDREN)**

**Name of field worker:** __________________________________________

**Area/Area code:** _______________________________________________

**Urban/Rural:** ________________________________________

**Household Number/Participant Number:** __________________________

**Tick the correct response to the following questions**

1. Does your child have a health card?
2. No (go to #2)
3. Yes (Use information on the card where applicable)
4. Where was he/she born?
5. Home
6. Hospital
7. Traditional birth attendant
8. Other
9. Was your child weighed at birth?
10. No
11. Yes, and if so how much did he/she weigh _________

Now we are going to measure your child’s height, weight and arm to see how well he/she is growing. Do you want to continue with the assessments? (*Discontinue if mother refuses*)

|  | **Age (months)/**  **gender** | **Weight (Kilograms)** | | | | **Height (metres)** | | | | **MUAC (Centimetres)** | | | |
| --- | --- | --- | --- | --- | --- | --- | --- | --- | --- | --- | --- | --- | --- |
|  |  | 1st | 2nd | 3^rd^ | Mean | 1st | 2^nd^ | 3rd | Mean | 1st | 2nd | 3rd | Mean |
| Child 1 |  |  |  |  |  |  |  |  |  |  |  |  |  |
| Child 2 |  |  |  |  |  |  |  |  |  |  |  |  |  |
| Child 3 |  |  |  |  |  |  |  |  |  |  |  |  |  |
| Child 4 |  |  |  |  |  |  |  |  |  |  |  |  |  |
| Child 5 |  |  |  |  |  |  |  |  |  |  |  |  |  |

Interviewer’s signature: ______________________ Date: _____________________
